# Ribitol-Containing Wall Teichoic Acid of Tetragenococcus halophilus Is Targeted by Bacteriophage phiWJ7 as a Binding Receptor

**DOI:** 10.1128/spectrum.00336-22

**Published:** 2022-03-21

**Authors:** Takura Wakinaka, Minenosuke Matsutani, Jun Watanabe, Yoshinobu Mogi, Masafumi Tokuoka, Akihiro Ohnishi

**Affiliations:** a Manufacturing Division, Yamasa Corporation, Choshi, Japan; b NODAI Genome Research Center, Tokyo University of Agriculture, Tokyo, Japan; c Faculty of Food and Agricultural Sciences, Fukushima University, Fukushima, Japan; d Institute of Fermentation Sciences, Fukushima University, Fukushima, Japan; e Department of Fermentation Science, Faculty of Applied Bio-Science, Tokyo University of Agriculture, Tokyo, Japan; University of Pittsburgh School of Medicine

**Keywords:** bacteriophage, host receptor, wall teichoic acid, *Tetragenococcus halophilus*

## Abstract

Tetragenococcus halophilus, a halophilic lactic acid bacterium, is used in the fermentation process of soy sauce manufacturing. For many years, bacteriophage infections of *T. halophilus* have been a major industrial problem that causes fermentation failure. However, studies focusing on the mechanisms of tetragenococcal host-phage interactions are not sufficient. In this study, we generated two phage-insensitive derivatives from the parental strain *T. halophilus* WJ7, which is susceptible to the virulent phage phiWJ7. Whole-genome sequencing of the derivatives revealed that insertion sequences were transposed into a gene encoding poly(ribitol phosphate) polymerase (TarL) in both derivatives. TarL is responsible for the biosynthesis of ribitol-containing wall teichoic acid, and WJ7 was confirmed to contain ribitol in extracted wall teichoic acid, but the derivative was not. Cell walls of WJ7 irreversibly adsorbed phiWJ7, but those of the phage-insensitive derivatives did not. Additionally, 25 phiWJ7-insensitive derivatives were obtained, and they showed mutations not only in *tarL* but also in *tarI* and *tarJ*, which are responsible for the synthesis of CDP-ribitol. These results indicate that phiWJ7 targets the ribitol-containing wall teichoic acid of host cells as a binding receptor.

**IMPORTANCE** Information about the mechanisms of host-phage interactions is required for the development of efficient strategies against bacteriophage infections. Here, we identified the ribitol-containing wall teichoic acid as a host receptor indispensable for bacteriophage infection. The complete genome sequence of tetragenococcal phage phiWJ7 belonging to the family *Rountreeviridae* is also provided here. This study could become the foundation for a better understanding of host-phage interactions of tetragenococci.

## INTRODUCTION

Tetragenococcus halophilus is a Gram-positive, halophilic lactic acid bacterium that is used for the manufacturing of various salted foods, such as soy sauce and salted fish (1, 2). *T. halophilus* plays an important role in the lactic acid fermentation process of these products, and similar to cases in dairy products, bacteriophages (or “phages”) have been regarded as serious threats in industry. Previously reported bacteriophages that attack *T. halophilus* have narrow host ranges and rarely infect different strains (3). Higuchi et al. successfully generated phage-insensitive mutants from *T. halophilus* D10, but the mechanisms that altered phage susceptibility were not revealed (4). Recently, CRISPR sequences were found in the genomes of *T. halophilus* and were proposed as one of the defense systems against bacteriophages (5). However, the factors that determine the phage susceptibility of this species were not experimentally demonstrated.

Adsorption to host cells by bacteriophages is the first step that defines the host range. Host receptors on the cell surface are specifically recognized by their respective bacteriophages. Phage adsorption generally consists of a two-step process: a reversible binding phase and an irreversible binding phase (6). In the first step, bacteriophages reversibly adsorb to host cells, and they can still desorb from the cells and can move on the cell surface to search for an optimal site for irreversible binding. In the second step, phages irreversibly attach to the host receptor, after which they cannot be separated from the host cells. Irreversible binding triggers phage genome ejection into the host cell, which is followed by a series of events for infection. Host receptors involved in reversible binding and irreversible binding are not always the same. For instance, λ phage that infect Escherichia coli target the outer membrane protein OmpC for reversible binding and the maltose transporter protein LamB for irreversible binding (7). A few receptors of Gram-positive bacteria have been identified (8). Peptidoglycan is the main component of bacterial cell walls and is often involved in phage adsorption (9). Another important component of the cell walls in Gram-positive bacteria involved in phage adsorption is cell wall teichoic acid (WTA). In most cases, WTA consists of polymers of glycerol phosphate (Gro-P) or ribitol phosphate (Rbo-P) linked via phosphodiester bonds (called Gro-WTA and Rbo-WTA, respectively) and covalently linked to peptidoglycan. WTA is produced by many Gram-positive bacteria and usually has species- or strain-specific structural patterns both in main chain components and decoration residues (10, 11).

**FIG 1:**
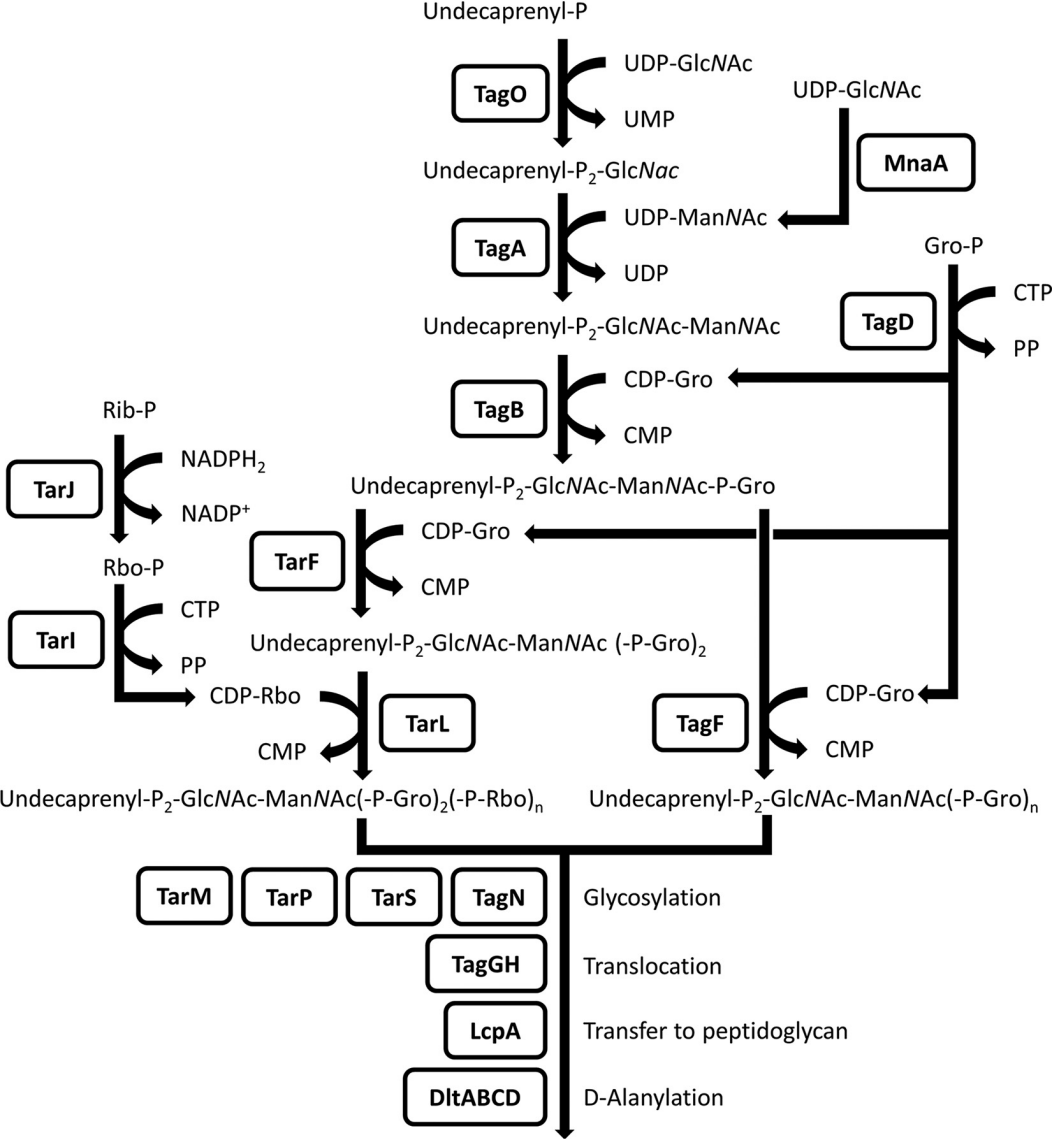
Biosynthetic pathways of Rbo- and Gro-WTA in S. aureus, adapted from previous reports (46–49). WTA synthesis is initiated with consecutive transfer of *N*-acetylglucosamine (Glc*N*Ac) phosphate and *N*-acetylmannosamine (Man*N*Ac) to the lipid carrier undecaprenylphosphate by TagO and TagA on the cytoplasmic side of the cell membrane. UDP-Man*N*Ac is generated from UDP-Glc*N*Ac by MnaA. In Gro-WTA synthesis, the primase TagB adds the first Gro-P to the disaccharide unit, and the polymerase TagF extends the Gro-P chain. The precursor CDP-glycerol is generated by TagD. In Rbo-WTA synthesis, the TagB reaction is followed by the addition of only one Gro-P unit by TarF. Subsequently, TarL polymerizes Rbo-P, and TarIJ is responsible for the generation of the precursor CDP-ribitol. Finally, the complete WTA polymers are translocated to the extracellular space by TagGH and are linked to the *N*-acetylmuramic acid residue of peptidoglycan by Lcp family proteins, primarily LcpA. The basic WTA polymers can be decorated with glycosyl residues and/or d-alanyl moieties; Rib-P, ribulose 5-phosphate; Rbo, ribitol; Gro, glycerol; P, phosphate; PP, diphosphate.

WTA biosynthesis genes are well understood in Staphylococcus aureus, namely, *tag* genes for Gro-WTA and *tar* genes for Rbo-WTA. To avoid confusion about the nomenclature, here, we use “*tag*” for the genes needed for Gro-WTA biosynthesis and for the genes commonly needed for both Gro-WTA and Rbo-WTA synthesis and use “*tar*” for the genes required only for Rbo-WTA synthesis, following the policy of Xia and Peschel (12). WTA synthetic pathways in S. aureus are shown in Fig. 1. The structures and synthetic pathways of WTA in *T. halophilus* have not been studied to date.

In this study, we generated two phage-insensitive derivatives from *T. halophilus* WJ7 susceptible to the virulent phage phiWJ7. Both of the derivatives have mutations in *tarL*, which is necessary for Rbo-WTA biosynthesis. Our subsequent experiments indicated that Rbo-WTA of WJ7 is a binding receptor for phiWJ7. The information provided here can represent a basis for dissecting the mechanisms of host-phage interactions of tetragenococci.

## RESULTS

### phiWJ7 is a member of the *Rountreeviridae*.

**FIG 2:**
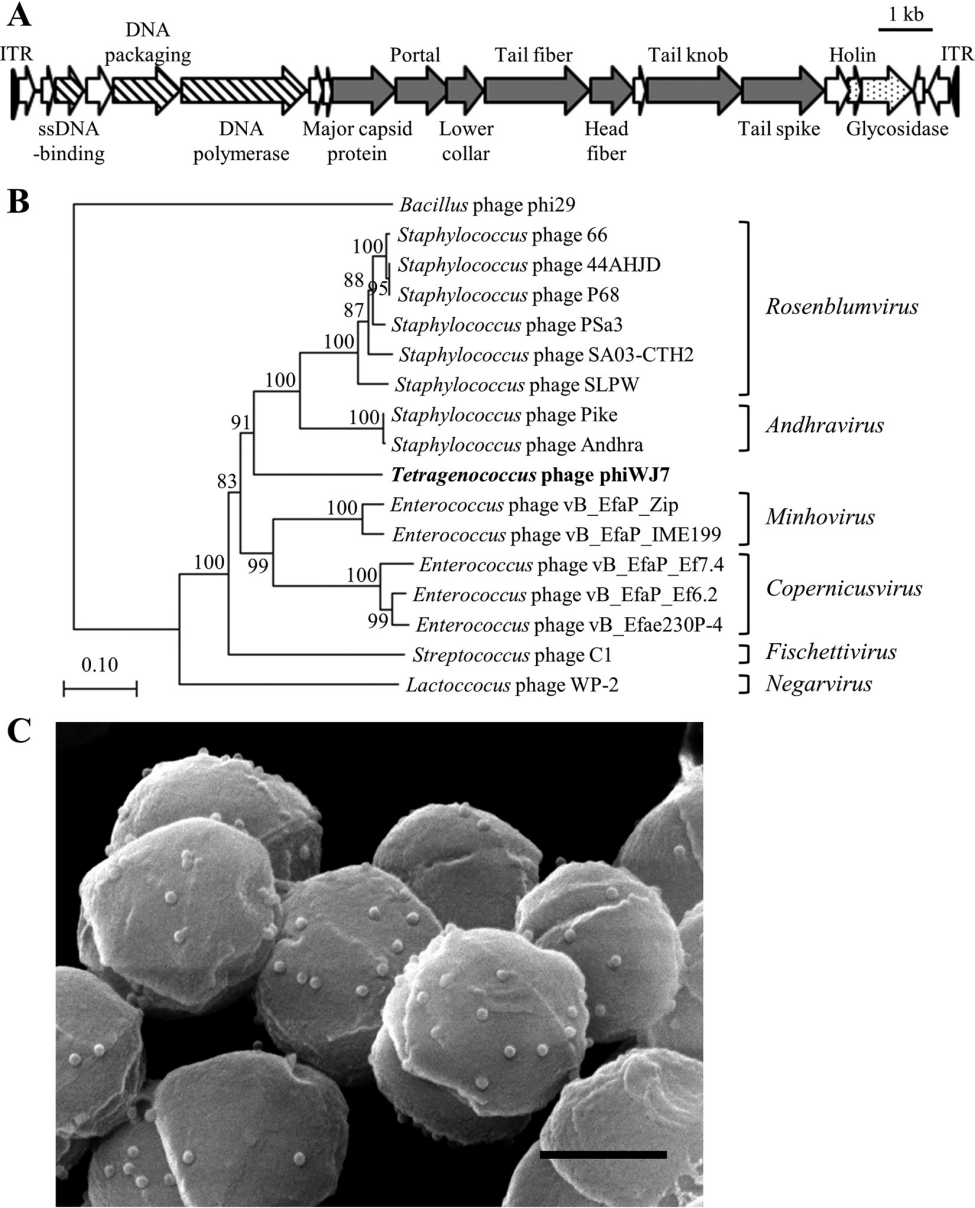
Characterization of phiWJ7. (A) Genetic map of the phiWJ7 genome. Arrows indicate the possible ORFs. Functional groups are categorized into patterns; striped, DNA replication and packaging; gray-shaded, structural proteins; dotted, bacterial lysis; blank, unknown. ITR indicates inverted terminal repeats and is marked by black triangles; ssDNA, single-stranded DNA. (B) Phylogeny of phiWJ7 and other *Rountreeviridae* phages based on the amino acid sequence relatedness of major capsid protein. The names of the six genera belonging to the family *Rountreeviridae* are denoted on the right side of each phage. *Bacillus* phage phi29 belongs to the family *Salasmaviridae*. (C) SEM image of WJ7 treated with phiWJ7. The black bar represents 500 nm.

PhiWJ7 lytic for *T. halophilus* WJ7 was isolated from soy sauce mash. The complete genome sequence of phiWJ7 was determined to be 17,405 bp in size with an average G+C content of 32.90% (accession number LC644557). The genome contains 21 possible open reading frames (ORFs) and more than 100-bp inverted terminal repeats (Fig. 2A). Detailed information about the ORFs is specified in Table S1 in the supplemental material. The ORFs whose functions were estimated were all arranged in the same direction and exhibited clustering of related functional genes. Some ORFs of phiWJ7 show similarity with genes of staphylococcal phages, such as SLPW, Andhra, SA03-CTH2, and PSa3. These phages belonged to the *Podoviridae* family of the *Caudovirales* order and have linear double-stranded DNA (dsDNA) genomes of 17,511 to 18,546 bp with 19 to 20 ORFs and long inverted terminal repeats (at least 81 bp) (13–15). Most staphylococcal phages characterized thus far belong to the order *Caudovirales* and had been classified into three families with different genome sizes: *Podoviridae* (16 to 18 kb), *Siphoviridae* (39 to 43 kb), and *Myoviridae* (120 to 140 kb) (16). Very recently, three new families, *Salasmaviridae*, *Rountreeviridae*, and *Guelinviridae*, were established (17). They include the phages previously assigned to the *Podoviridae* family. *Rountreeviridae* is mainly composed of Staphylococcus- and *Enterococcus*-infecting phages with genomes between 17 and 19 kb in size. The genome size and protein sequence homologies suggest the classification of phiWJ7 into *Rountreeviridae*. We aligned the sequences of the major capsid protein from phiWJ7 and other *Rountreeviridae* phages and compared the phylogenetic relationships. The results confirmed phiWJ7 classification into *Rountreeviridae* (Fig. 2B). Scanning electron micrograph (SEM) images of phiWJ7 bound to WJ7 displayed an isometric capsid of approximately 44 nm in diameter and hardly observable tails, which are the typical morphological characteristics of *Rountreeviridae* (Fig. 2C).

### Generation of phiWJ7-insensitive derivatives and other bacteriophages lytic for WJ7.

**FIG 3:**
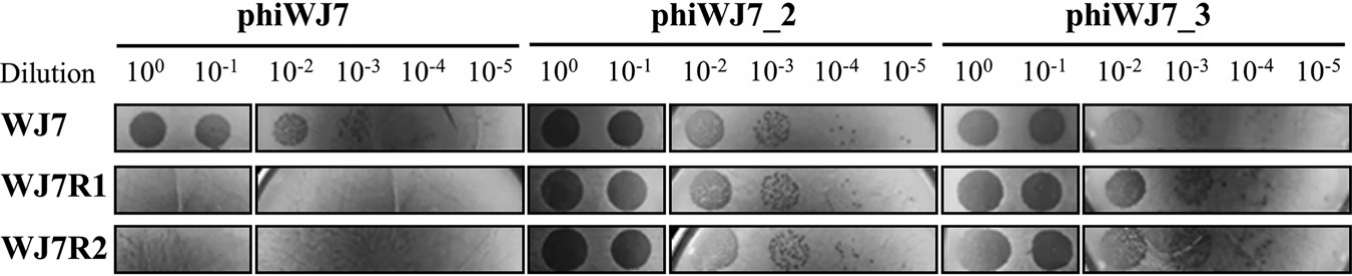
Phage susceptibility of the host strains toward phiWJ7, phiWJ7_2, and phiWJ7_3. Each phage specimen was diluted and spotted on each host strain indicated on the left. The various dilutions of a phage were spotted on the same plate of a strain, although it is divided.

Spontaneous phiWJ7-insensitive derivatives WJ7R1 and WJ7R2 were selected from the parental strain WJ7 as colonies growing in the presence of phiWJ7 on LA13 agar plates. PhiWJ7 forms clear plaques to WJ7, but, even with 10^9^ PFU phiWJ7, no plaques were formed to these derivatives (Fig. 3; Table S2). However, they were infected with other bacteriophages, phiWJ7_2 and phiWJ7_3, which were also isolated from soy sauce mash. PhiWJ7_2 made similar plaques to phiWJ7 in clearance and size and infected WJ7 and its derivatives. Interestingly, phiWJ7_3 formed a smaller size and a ower number of plaques to WJ7 than to WJ7R1 and WJ7R2, which suggests that the derivatives were more vulnerable to phiWJ7_3 instead of acquiring resistance to phiWJ7. According to the genome sequence, phiWJ7_2 belongs to the family *Siphoviridae* (unpublished data), and phiWJ7_3 is not characterized yet.

### Detection of mutational and insertion sequence insertion sites in the two adapted genomes.

**FIG 4:**
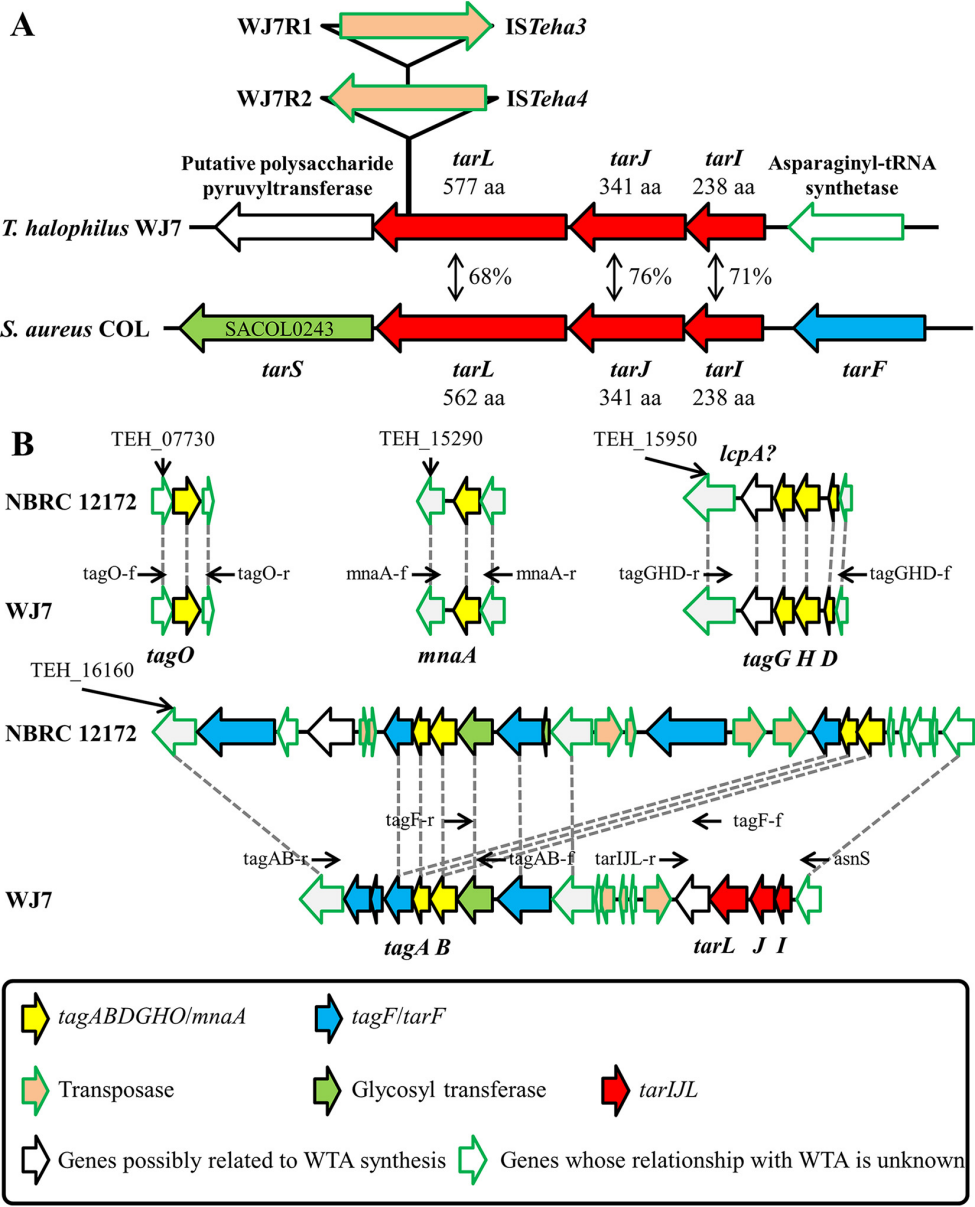
WTA synthesis genes of WJ7. (A) Schematic representation of *tarIJL* in *T. halophilus* WJ7 and S. aureus COL and the location of ISs transposed into *tarL* in WJ7R1 and WJ7R2. Percentage shows the amino acid identities between *T. halophilus* and S. aureus. The derivative names and IS names are indicated on the left and right of the arrows that represent the putative transposase, respectively. The locus tags of the WTA synthesis genes in COL and NBRC 12172 are designated in Table S4 in the supplemental material; aa, amino acids. (B) Chromosomal organization of the four loci harboring the genes involved in WTA synthesis in the NBRC 12172 and WJ7 genomes. Homologous ORFs with amino acid sequence identities above 90% are connected with gray dotted lines, except for transposases. The black arrows above each ORF indicate the position at which the primers were designed.

To investigate the mechanism that altered the phage susceptibility of the derivatives, whole-genome sequences of WJ7R1 and WJ7R2 were analyzed, and the mutation sites of the derivatives were clarified by genome mapping analysis. A single base insertion was commonly observed in both derivatives but not in ORFs (178 bp upstream of an ORF; locus tag WJ7_08810). We considered that this mutation had presumably occurred during the culture of the parent strain and was unlikely to contribute to the phenotypic change. Only one other mutation was found in each derivative. Both WJ7R1 and WJ7R2 contained a transposed insertion sequence (IS) in the same ORF (WJ7_16330), IS*Teha3* in WJ7R1 and IS*Teha4* in WJ7R2 (Fig. 4A). In a previous study, we found that the transposition of these IS*4* family ISs plays an important role in the disruption of the arginine deiminase system in *T. halophilus* NBRC 12172 (18). Transposition activities of the ISs in WJ7 were also demonstrated here, and they targeted a AT-rich 7-bp sequence on the ORF, which is a typical feature of these ISs (Table S3). The amino acid sequence of this mutated ORF showed 68% similarity with poly(ribitol phosphate) polymerase (TarL) of S. aureus, and two ORFs located upstream of *tarL* also showed high amino acid similarity with ribitol-5-phosphate cytidylyltransferase (TarI) and ribulose 5-phosphate reductase (TarJ) of S. aureus. TarIJL is responsible for the biosynthesis of Rbo-WTA (Fig. 1).

### *In silico* analysis of the WTA biosynthesis genes in *T. halophilus*.

Since IS transpositions independently occurred into *tarL* in the two phage-insensitive derivatives, it is most likely that the disruption of the Rbo-WTA synthetic pathway altered phage susceptibility. However, there has not been systematic consideration of WTA synthesis genes in *T. halophilus* before. Therefore, we first carried out *in silico* analysis of the WTA biosynthetic pathways in *T. halophilus* NBRC 12172, whose complete genome was deposited in the DDBJ database (accession number AP012046). The genes involved in WTA synthesis were searched by blastp using the amino acid sequences of the genes in S. aureus COL as queries. As a result, the genome of NBRC 12172 contains homologs of *tagO*, *mnaA*, *tagADB*, *tarF*, and *tagGH*, which are scattered over four chromosomal loci (Fig. 4B; Table S4). There are five homologs of *tarF*, one of which might be *tagF* since *tarF* and *tagF* are homologous to each other (19). NBRC 12172 possesses three Lcp family proteins, and one of them is assumed to be mainly responsible for the transfer of WTA to peptidoglycan; TEH_15960, adjoining *tagG*, is the most likely candidate. Thus, it was estimated that NBRC 12172 possesses the gene set for Gro-WTA synthesis; however, it does not contain *tarIJL* required for Rbo-WTA synthesis. The counterparts of these genes were searched in the genome of WJ7. WJ7 was also estimated to contain the gene set for Gro-WTA synthesis, and *tarIJL* was located in the same chromosomal locus as *tagAB* and *tagF*/*tarF* (Fig. 4B). In conclusion, it was estimated that WJ7 is endowed with gene sets for both Gro-WTA and Rbo-WTA synthesis. We compared the *tagAB*-containing locus with three other strains, YA5, YA163, and YG2, for which draft genome sequences are available (5). The three strains did not contain *tarIJL* in their genomes, similar to NBRC 12172, and all of them were insusceptible to phiWJ7 (Fig. S1).

### Alditol detection from WTA of WJ7 and WJ7R1.

**FIG 5:**
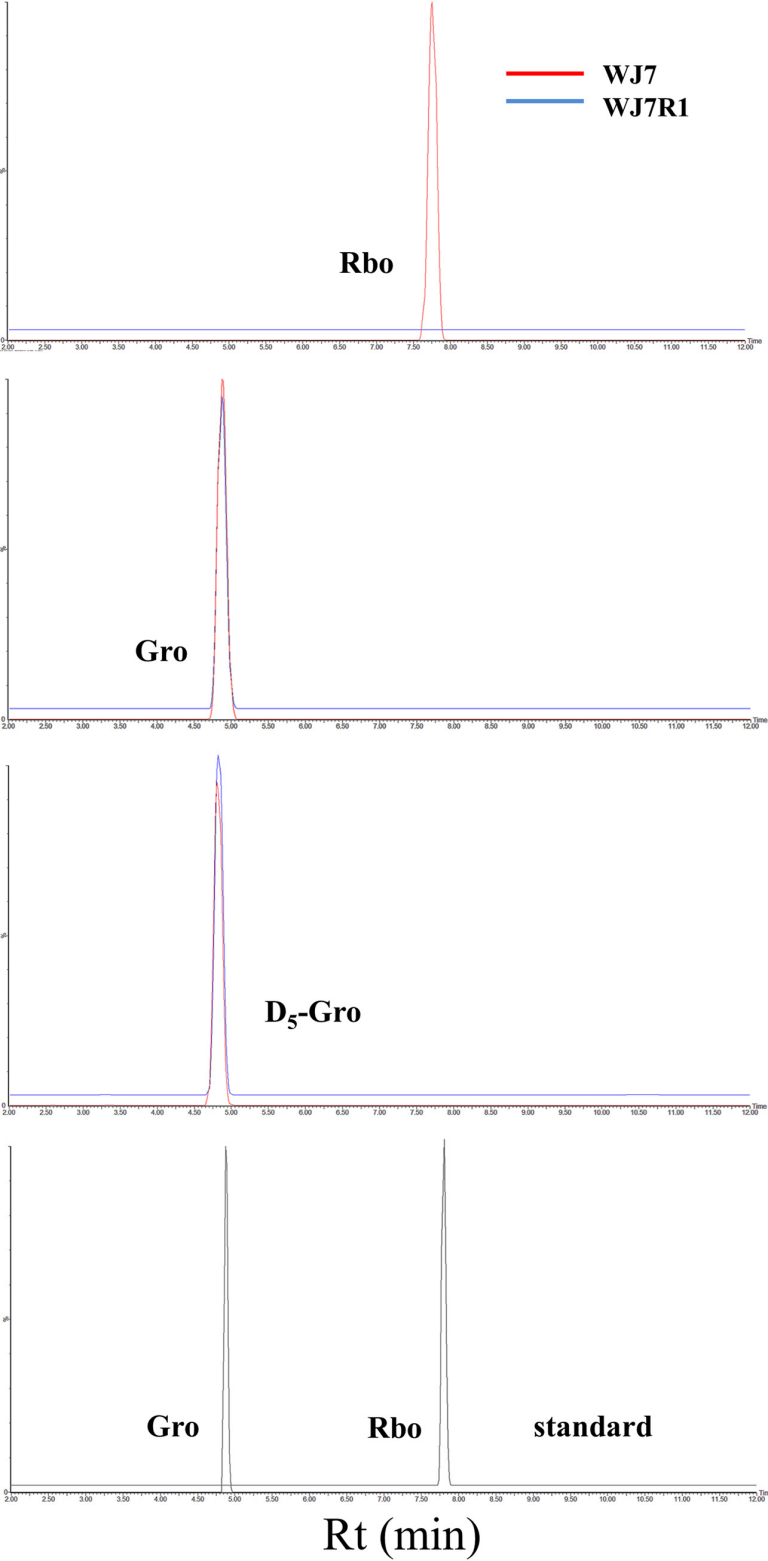
Extracted ion chromatogram of the benzoyl derivatives of Rbo (*m*/*z* 690.23), Gro (*m*/*z* 422.16), and D_5_-Gro (1,1,2,3,3-D_5_-glycerol; *m*/*z* 437.19) in the cell wall samples of WJ7 and WJ7R1. Extracted ion chromatograms of Gro and Rbo in the reference standard sample are also shown; Rt, retention time.

Since WJ7 possessed gene sets for Gro-WTA and Rbo-WTA synthesis and the Rbo-WTA synthesis gene was disrupted in WJ7R1, we assessed the presence of Rbo and Gro in extracted WTA from WJ7 and WJ7R1. WTA extracted from the cell walls by trichloroacetic acid (TCA) was hydrolyzed and derivatized with benzoyl chloride followed by analysis with liquid chromatography (LC)/mass spectrometry (MS). Derivatized Rbo and Gro were detected as ammonium adducts, and their exact masses were *m*/*z* 690.23 and 422.16, respectively. The derivatization efficiencies of the samples were normalized by comparing the peak intensities of D_5_-glycerol. Extracted ion chromatograms of *m*/*z* 690.23 showed a clear peak only in the hydrolysate sample of WJ7 at the same elution time corresponding to the standard Rbo peak (Fig. 5). We also confirmed that extracted ion chromatograms of *m*/*z* 422.16 showed clear peaks with almost the same intensities in both the WJ7 and WJ7R1 samples at the elution time of standard Gro. These results suggest that WJ7 produces Rbo-WTA, but the phage-insensitive derivative WJ7R1 does not, while both strains produce Gro-WTA at almost the same level.

### Bacteriophage adsorption by cell walls.

**FIG 6:**
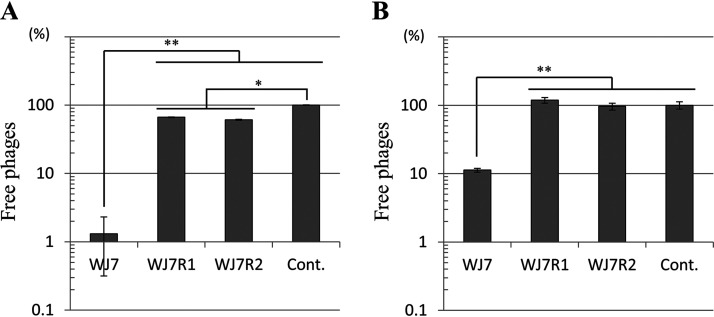
PhiWJ7 adsorption to the cell walls of WJ7 and the derivatives. Free phage titers were calculated as percentages of the control (without cell walls); Cont., control sample. Data are expressed as the mean with error bars representing ± standard deviation (SD; *n* = 3). Bars with asterisks are significantly different by Tukey’s multiple-comparison test (*, *P* < 0.05; **, *P* < 0.01). (A) Free phages represent total adsorption (the sum of phages reversibly and irreversibly adsorbed). (B) Free phages after 100-fold dilution of the samples represent irreversible adsorption.

The WTA of Gram-positive bacteria could be the host receptor for bacteriophage adsorption (8). Therefore, we performed a phage adsorption assay with cell walls of WJ7 and the two derivatives. The cell walls were mixed with phiWJ7, and the bound phages were removed by centrifugation. The phage adsorption was calculated from the free phage titer of the supernatant. Almost 99% of phiWJ7 adsorbed to the cell walls of WJ7 (Fig. 6A), which verifies the presence of a binding receptor for phiWJ7. Yet, 34 to 39% of phiWJ7 adsorbed to WJ7R1 and WJ7R2, which indicates that a binding receptor for phiWJ7 is impaired in the derivatives, but the derivatives still adsorb to phiWJ7. Next, we observed irreversible phage adsorption by diluting the cell wall-phage mixtures 100-fold prior to centrifugation, a procedure that releases reversibly bound phages (20). After the dilution, the number of free phages was increased. However, 89% of phiWJ7 still adsorbed to the cell walls of WJ7, whereas phiWJ7 that adsorbed to WJ7R1 and WJ7R2 was totally released (Fig. 6B), which demonstrates that WJ7 contains the irreversible binding receptor for phiWJ7, and the derivatives lack this receptor. Considering these results along with the fact that the derivatives have IS transposition in *tarL* and that WJ7R1 lacks Rbo in the extracted WTA, it was estimated that Rbo-WTA is an indispensable binding receptor for phiWJ7. Since the derivatives were reversibly adsorbed by phiWJ7, there must be another cell wall component than Rbo-WTA involved in primary reversible attachment.

### Additional acquisition of phiWJ7-insensitive derivatives and identification of the mutation sites.

Suppose that Rbo-WTA is an indispensable binding receptor for phiWJ7, and mutants that lack any genes participating in Rbo-WTA synthesis are expected to be acquired as phiWJ7-insensitive derivatives. When these genes are disrupted by IS transposition, the mutants could easily be found by comparing the length of PCR-amplified DNA fragments containing the disrupted genes. Thus, we acquired additional 25 phiWJ7-insensitive derivatives (WJ7R3 to WJ7R27) in the same way as WJ7R1 and WJ7R2 were obtained and amplified the DNA region covering the WTA synthesis genes with the primers indicated in Fig. 4B and Table 1. Surprisingly, 21 of 25 derivatives displayed elongated PCR products in the *tarL*-containing region compared with the product amplified from the parental strain of WJ7, and none of the derivatives showed apparent different lengths of PCR products covering other regions (data not shown). DNA sequence analyses revealed that the 21 derivatives had IS transposition into *tarI*, *tarJ*, or *tarL* (Fig. S2 and Table S3). These 21 derivatives showed the same phage susceptibility as WJ7R1: insensitive to phiWJ7, sensitive to phiWJ7_2 the same as WJ7, and more sensitive to phiWJ7_3 than WJ7 (Fig. S3). Among the remaining four derivatives, WJ7R12 and WJ7R24 were less sensitive to phiWJ7_2 and phiWJ7_3 than WJ7R1. We considered that these two derivatives have different mechanisms of altered phage susceptibility than WJ7R1. The other two strains, WJ7R4 and WJ7R18, showed the same phage susceptibility as WJ7R1. Hence, we analyzed the DNA sequence of *tarIJL* and found one base substitution in *tarJ* of WJ7R4 causing the amino acid replacement Y102C and a 102-bp deletion from *tarJ* to *tarL* in WJ7R18 (Fig. S2). In conclusion, all 23 additionally acquired derivatives that displayed the same phage susceptibility as WJ7R1 contained mutations in *tarIJL*. Lack of TarIJ also disrupts Rbo-P polymerization by abstaining to provide the donor substrate CDP-ribitol (Fig. 1).

**TABLE 1:** Primers list

Primer name	Sequence (5′→3′)
WJ7contig68	GAGGAACTACATCATAATTAAATAACTCGC
asnS	CGTTTATTGAATCGGATTTATCCG
tagOf	TGAATGCTTCGATGGAAGAG
tagOr	TCCCATTCGGAATCGC
mnaAf	GGCAAAGACCTAGATTCTATCCG
mnaAr	CAATGCTTGAAAGTCAATCGTC
tagGHDr	GCGTCGAACTTGCGG
tagGHDf	GGTTTAGACGTATGGGAACATGC
tagABr	TCTTCTGATTGGTTCTTGTCACTC
tagABf	AGGTGAATATCGCTCAAGGG
tagFr	GCCATTCATCGCCTAACTG
tagFf	CAGCGCCACTGGACAC
tarIJLr	TCGTAACCTTGTAGCCTATCGG

## DISCUSSION

Bacteriophages are a significant concern for fermentation industries. Industries have dealt with this problem for many years and are now implementing a variety of approaches to control phages, such as factory design changes, sanitation improvement, sterilization of raw materials, and culture rotation. However, since soy sauce is generally manufactured in imperfectly closed facilities, it is difficult to completely exclude bacteriophages from factories. *T. halophilus* severely affects the quality of soy sauce products not only by lactic acid production but also by other traits. For instance, most *T. halophilus* strains produce citrulline as a metabolic intermediate of the arginine deiminase system. Citrulline is reported as the main precursor of the potential carcinogen ethyl carbamate in soy sauce (21). Normally, citrulline is converted to ornithine inside bacterial cells, but when the cells are lysed by bacteriophages, it is discharged outside the cells and makes ethyl carbamate in soy sauce (18). Thus, phage infection of fermentation starters remains one of the most common causes of incomplete fermentation and product downgrading.

Extensive efforts have been made to acquire many strains suitable for starters as a preparation for the appearance of bacteriophages (4). The use of phage-insensitive derivatives is one of the solutions, but a mutated strain resistant to a particular phage is not necessarily resistant to other phages, as our results clearly demonstrated, which highlights the importance of understanding the host-phage interaction mechanisms. Some bacterial antiphage defense systems are known, such as restriction/modification (22), toxin/antitoxin (23), and CRISPR-Cas systems (24). In addition, cell surface structure alterations are an important phage defense mechanism. Genetic mutations affecting the structures or expression of phage-binding receptors prevent the adsorption of a particular phage and change phage susceptibility (25, 26). Previous studies identified a few phage receptor molecules of Gram-positive bacteria, such as cell membrane-associated proteins, peptidoglycan, lipoteichoic acid, and WTA (9, 27, 28). Here, we demonstrated that phiWJ7 requires Rbo-WTA as a host receptor for irreversible binding and that WJ7 becomes resistant to phiWJ7 by mutating Rbo-WTA synthesis genes. Many staphylococcal *Rountreeviridae* phages also use Rbo-WTA as a binding receptor (16).

The difference in WTA structures is established as one of the determining factors of phage susceptibility in *T. halophilus*. Staphylococcal phages usually infect strains with common WTA structures, whereas previously studied tetragenococcal phages showed narrow host ranges and were reported to be almost “strain specific” (3). It was estimated that phiWJ7 recognizes its hosts by combining Rbo-WTA and the still-unknown reversible binding receptor. The receptors of phiWJ7_2 and phiWJ7_3 are not known, but they are not Rbo-WTA. Accordingly, other receptors in addition to Rbo-WTA should also play an important role in host recognition by tetragenococcal phages. In this study, however, most of the derivatives obtained for phiWJ7 resistance contained a mutation in *tarIJL*. The reason why the derivatives devoid of reversible binding receptors for phiWJ7 could scarcely be acquired is perhaps because the reversible binding receptor is not essential for infection of some phages and only allows their rapid recognition of irreversible binding receptors (20). Another possibility is that the reversible binding receptor for phiWJ7 is indispensable for the cell viability of WJ7. WJ7 becomes more vulnerable to phiWJ7_3 when Rbo-WTA synthesis genes are disrupted. The reason is unclear, but possibly Rbo-WTA masks the receptor for phiWJ7_3 and prevents phiWJ7_3 adsorption, or the expression level of the receptor for phiWJ7_3 is changed as Rbo-WTA synthesis is impaired.

The *in silico* analysis of the WJ7 genome and the chemical analysis of the WTA extracted from WJ7 suggested the presence of both Gro-WTA and Rbo-WTA, but their structures and synthetic pathways are not fully clarified. In S. aureus, *tarIJL* is located upstream of *tarS* encoding a glycosyltransferase that attaches β-*N*-acetylglucosamine (β-Glc*N*Ac) residues to Rbo-WTA (29), while *tarIJL* of WJ7 is followed by a putative polysaccharide pyruvyltransferase (Fig. 4A). Nocardiopsis metallicus actually produces Rbo-WTA with pyruvate residues (30). Further research is necessary to fully understand the structures and biosynthetic pathways of WTA in *T. halophilus*.

In the context of host-phage interactions, WTA is often regarded as the host receptor for phage adsorption, and we added additional evidence to show this. Additionally, various roles of WTA were proposed, such as protection of pathogens from host defense and antibiotics, regulation of cell division, ion homeostasis, adhesion, and biofilm formation (10). The WTA function for *T. halophilus* is unclear. The deletion of *tagO*, whose product catalyzes the first step in WTA synthesis, results in a complete lack of WTA in cell walls and is not lethal for S. aureus (31). Nevertheless, *tagO* mutants were not obtained in this study, suggesting that WTA may play some important roles in *T. halophilus* and that *tagO* mutants may be severely compromised, as in Bacillus subtilis (32). In S. aureus, the disruption of late-stage Rbo-WTA synthesis genes, such as *tagBDGH* and *tarFIJL*, is lethal, probably because the accumulation of undecaprenol-linked intermediates depletes lipid carriers that are also needed for peptidoglycan synthesis (33). In *T. halophilus*, *tarIJL* mutants were obtained, and under laboratory conditions, WJ7R1 and WJ7R2 were not different in growth rate and morphology from WJ7 (Fig. S4 in the supplemental material). We hypothesized that this is because *tarIJL* mutants do not accumulate the intermediates but use them for Gro-WTA production. Lactiplantibacillus plantarum activates Rbo-WTA synthesis genes when *tagF* is impaired (34).

Despite notable industrial concern, little is known about tetragenococcal phages. Only three phages, Φ7116 (*Myoviridae*), ΦD-86, and ΦD10 (both *Siphoviridae*), have been characterized thus far, but their genomic sequences have not been published (3, 4). We present the complete genome sequence of phiWJ7 belonging to the *Rountreeviridae* family. The phiWJ7 genome resembles those of some staphylococcal *Rountreeviridae* phages in genome size, protein sequence homology, and the presence of long inverted terminal repeats. PhiWJ7 presumably evolved from a common ancestor of such phages. We compared the genome of phiWJ7 with that of Staphylococcus phage P68 (Fig. 2B) in which the virion-constituting proteins were identified, and the virion structures were elucidated (35). Among 10 structural proteins of P68, six showed similarity with the proteins of phiWJ7 by blastp search (Fig. S5A). P68 binds to Rbo-WTA, and the tail fiber is responsible for the adsorption. The ORF12 of phiWJ7 showed 34% amino acid similarity to the tail fiber of P68, which suggests that ORF12 of phiWJ7 is involved in the interaction with Rbo-WTA. The head fiber of P68 is considered to function in the primary reversible attachment of the phages to cells. Although none of the proteins of phiWJ7 showed similarity with the entire head fiber of P68, the N-terminal part of ORF13 of phiWJ7 was similar to that part of the P68 head fiber (Fig. S5B). The head fiber of P68 contains an N-terminal capsid-binding domain and the C-terminal receptor-binding domain. Hence, ORF13 of phiWJ7 is likely to code the head fiber but bind to a different receptor from P68.

In this study, IS*Teha3*, IS*Teha4*, and IS*Teha5* played important roles in the disruption of the Rbo-WTA synthetic pathway, which emphasizes the contribution of ISs to mutations and the evolution of *T. halophilus.* First, we discovered the transposition of these ISs in UV-irradiated derivatives (18), but it was shown here that transposition occurred spontaneously without UV irradiation. Unfortunately, the lack of an established transformation technique in *T. halophilus* makes it difficult to adopt cloning strategies to analyze gene functions. In this study, we adopted a strategy utilizing intrinsic ISs, which is similar to a transposon mutagenesis system. It is advantageous that the transposition of ISs can be easily detected by PCR amplification if the transposition site is predicted and that the mutant strains do not contain foreign genes so they can immediately be available in a food-grade form. However, the disadvantage is that the transposition is uncontrollable in frequency and location. The ISs used here preferentially target AT-rich regions and are relatively difficult to insert into shorter ORFs. Since a number of genes other than *tarIJL* contribute to Rbo-WTA synthesis and their total length is sufficiently long compared with *tarIJL*, we think the unsuccessful acquisition of mutants in which such genes were disrupted by ISs is likely to be caused by the reason described above. However, when expecting short genes without sufficient AT-rich regions to be disrupted, the necessity to consider the difficulty of IS insertion increases. We hope to understand the nature of these ISs better and hope that the technique will be developed in the future to obtain derivatives in which a particular gene is impaired by the ISs. Such a technique will contribute to the development of efficient strategies against bacteriophage infections.

## MATERIALS AND METHODS

### Bacterial strains, bacteriophages, media, and culture conditions.

*T. halophilus* WJ7 was kept in our laboratory (36). The derivatives derived from WJ7 were obtained as described below. Bacteriophages lytic for WJ7 were isolated from soy sauce mash using WJ7 as the host strain. All bacterial strains and bacteriophages used in this study are summarized in Table 2. *T. halophilus* was cultured in MRS-10 or LA13 medium. MRS-10 is lactobacilli MRS broth (Difco, Detroit, MI) supplemented with 10% NaCl. LA13 medium contained 1% polypeptone, 0.4% yeast extract, 1% KH_2_PO_4_, 12% NaCl, 0.5% glucose, 5% soy sauce (Yamasa, Choshi, Japan), and 1.5% agar for agar plates (37). Liquid media were incubated at 30°C without stirring, and agar plates were incubated at the same temperature with an AnaeroPack (Mitsubishi Gas Chemical, Tokyo, Japan) in a hermetically sealed box.

**TABLE 2:** Bacterial strains and bacteriophages used in this study

Bacteria strains and bacteriophages	Description and genotype	Source or reference
Tetragenococcus halophilus		
NBRC 12172	Insensitive for phiWJ7.	National Bio Resource Center
YA5	Insensitive for phiWJ7.	18
YA163	Insensitive for phiWJ7.	36
YG2	Insensitive for phiWJ7.	36
WJ7	Sensitive for phiWJ7.	36
WJ7R1	Insensitive for phiWJ7. *tarL*::IS*Teha3*	WJ7 derivative generated in this study.
WJ7R2	Insensitive for phiWJ7. *tarL*::IS*Teha4*	WJ7 derivative generated in this study.
WJ7R3	Insensitive for phiWJ7. *tarL*::IS*Teha4*	WJ7 derivative generated in this study.
WJ7R4	Insensitive for phiWJ7. *tarJ*.A305G	WJ7 derivative generated in this study.
WJ7R5	Insensitive for phiWJ7. *tarI*::IS*Teha3*	WJ7 derivative generated in this study.
WJ7R6	Insensitive for phiWJ7. *tarJ*::IS*Teha4*	WJ7 derivative generated in this study.
WJ7R7	Insensitive for phiWJ7. *tarI*::IS*Teha4*	WJ7 derivative generated in this study.
WJ7R8	Insensitive for phiWJ7. *tarJ*::IS*Teha4*	WJ7 derivative generated in this study.
WJ7R9	Insensitive for phiWJ7. *tarL*::IS*Teha4*	WJ7 derivative generated in this study.
WJ7R10	Insensitive for phiWJ7. *tarJ*::IS*Teha4*	WJ7 derivative generated in this study.
WJ7R11	Insensitive for phiWJ7. *tarI*::IS*Teha4*	WJ7 derivative generated in this study.
WJ7R12	Insensitive for phiWJ7.	WJ7 derivative generated in this study.
WJ7R13	Insensitive for phiWJ7. *tarL*::IS*Teha4*	WJ7 derivative generated in this study.
WJ7R14	Insensitive for phiWJ7. *tarL*::IS*Teha4*	WJ7 derivative generated in this study.
WJ7R15	Insensitive for phiWJ7. *tarL*::IS*Teha4*	WJ7 derivative generated in this study.
WJ7R16	Insensitive for phiWJ7. *tarI*::IS*Teha4*	WJ7 derivative generated in this study.
WJ7R17	Insensitive for phiWJ7. *tarL*::IS*Teha4*	WJ7 derivative generated in this study.
WJ7R18	Insensitive for phiWJ7. *tarJ.993_tarL.35*del	WJ7 derivative generated in this study.
WJ7R19	Insensitive for phiWJ7. *tarJ*::IS*Teha4*	WJ7 derivative generated in this study.
WJ7R20	Insensitive for phiWJ7. *tarJ*::IS*Teha4*	WJ7 derivative generated in this study.
WJ7R21	Insensitive for phiWJ7. *tarJ*::IS*Teha4*	WJ7 derivative generated in this study.
WJ7R22	Insensitive for phiWJ7. *tarL*::IS*Teha4*	WJ7 derivative generated in this study.
WJ7R23	Insensitive for phiWJ7. *tarL*::IS*Teha4*	WJ7 derivative generated in this study.
WJ7R24	Insensitive for phiWJ7.	WJ7 derivative generated in this study.
WJ7R25	Insensitive for phiWJ7. *tarI*::IS*Teha4*	WJ7 derivative generated in this study.
WJ7R26	Insensitive for phiWJ7. *tarI*::IS*Teha4*	WJ7 derivative generated in this study.
WJ7R27	Insensitive for phiWJ7. *tarL*::IS*Teha5*	WJ7 derivative generated in this study.
Bacteriophage		
phiWJ7	Lytic for WJ7. Not lytic for WJ7R1 and WJ7R2.	Isolated from soy sauce mash.
phiWJ7_2	Lytic for WJ7, WJ7R1, and WJ7R2.	Isolated from soy sauce mash.
phiWJ7_3	Lytic for WJ7, WJ7R1, and WJ7R2.	Isolated from soy sauce mash.

### Manipulation of bacteriophages and generation of phage-insensitive derivatives.

Uchida and Kanbe developed a detection method for tetragenococcal phages by spreading host-phage mixtures on agar plates with a cell spreader, since the usual soft agar overlay technique was not successful (3). We used this method with a little modification. Before the plaque-forming assay, the indicator strains cultured in MRS-10 to the stationary phase were sonicated by a sonicator (AUS-01; CHO-ONPA KOGYO, Tokyo, Japan) for 30 s to produce homogenous cell suspensions because some strains of *T. halophilus* form cell clusters (36). The suspension was diluted 6 times with 10% saline, and 600 μL of the diluted suspension was mixed with the phage specimen. The host-phage mixture was spread on the whole surface of an LA13 plate (diameter 85 mm) by tilting the plate without using a cell spreader to prevent traces of a cell spreader from disturbing plaque detection. The plate was dried on a clean bench and incubated for 3 days to form plaques. For the phage spot assay, the host suspension was spread in advance, and 10 μL of phage specimen was spotted afterward. If necessary, phages were collected from plaques with 10% saline, and the lysate was filtered through a polyethersulfone membrane filter (0.2 μm; Advantec, Tokyo, Japan) after removal of cell debris by centrifugation. The lysate was stored at −80°C as a stock suspension with 16% (vol/vol) glycerol. To obtain spontaneous phage-insensitive derivatives, the culture of the parental strain (approximately 10^6^ CFU) was spread with >10^7^ PFU of phages on LA13 plates, and the surviving colonies were isolated.

### Scanning electron microscopy.

WJ7 was mixed with phiWJ7, and the mixture was incubated for 1 h at room temperature. Fixation was performed by immersing the specimens in 2.5% glutaraldehyde. Fixed specimens were stepwise dehydrated in a series of ethanol solutions (50, 70, 90, 100, and 100%) in 15-min steps. Dehydrated specimens were lyophilized in *tert*-butyl alcohol. Afterward, the specimens were coated with a thin Pt layer using an autofine coater JEC-3000FC (JEOL, Ltd., Tokyo, Japan) and observed with a JSM-IT500HR InTouchScope scanning electron microscope (JEOL, Ltd.). Scanning electron microscopy was performed at 15 kV, and the samples were observed at a working distance of 11.0 mm.

### DNA preparation and genome sequencing.

Genomic DNA from *T. halophilus* WJ7R1 and WJ7R2 was isolated using the DNeasy PowerSoil Pro kit (Qiagen, Hilden, Germany) and QIAcube, an automated system (Qiagen). Genomic DNA from bacteriophage phiWJ7 was isolated using a phage DNA isolation kit (Norgen Biotek, Thorold, Canada). The quantity and purity of the genomic DNA were assessed using a Qubit dsDNA broad-range (BR) assay kit (Thermo Fisher Scientific, Waltham, MA) and NanoDrop 1000 spectrophotometer (Thermo Fisher Scientific). A genomic DNA library was prepared using the Illumina Nextera DNA Flex library prep kit (Illumina, San Diego, CA) according to the manufacturer’s instructions. Whole-genome sequencing of WJ7R1, WJ7R2, and phiWJ7 was performed using the Illumina MiSeq sequencing platform with a paired-end sequencing strategy (2 × 300 bp). Adapter sequences and low-quality regions in the Illumina reads were trimmed using Trim Galore! v.0.6.4 with default parameters (https://www.bioinformatics.babraham.ac.uk/projects/trim_galore/).

### Genome mapping analysis and mutation search of the derivatives.

The previously published genome sequence of the wild-type strain *T. halophilus* WJ7 (GenBank accession numbers BLRM01000001 to BLRM01000113) was used as the reference sequence for our genome mapping analysis (5). The Illumina sequence reads of these strains were mapped to the reference sequence using BWA with default parameters (38). All single nucleotide polymorphisms (SNPs) and indels were detected using the Genome Analysis Toolkit (GATK) (39, 40). To detect the transposon insertion site of adapted genomes, breseq v.0.31.0 was used with default parameters (41). To ascertain the mutations detected in the adapted strain, the DNA regions were amplified from genomic DNA of WJ7, WJ7R1, and WJ7R2 using the primers WJ7contig68 and asnS (Table 1) with DNA polymerase KOD FX Neo (Toyobo, Osaka, Japan) under the optimal conditions recommended by the manufacturer. The PCR products were purified using the Wizard SV gel and PCR clean-up system (Promega, Madison, WI), and the resulting DNA fragment was analyzed by a commercial DNA sequencing service (Fasmac, Atsugi, Japan/Eurofins Genomics, Tokyo, Japan). The primer sets tagOf-tagOr, mnaAf-mnaAr, tagGHDr-tagGHDf, tagABr-tagABf, tagFr-tagFf, and asnS-tarIJLr were used to amplify the DNA region covering WTA synthesis genes to detect the IS insertion in the additionally acquired derivatives WJ7R3 to WJ7R27 (Table 1 and Fig. 4B). If necessary, the resulting DNA fragment was analyzed by a DNA sequencing service (Fasmac/Eurofins Genomics).

### *De novo* assembly of phage genomes.

To remove the contaminated host genome sequence, read data were mapped onto the WJ7 genome (39, 40). Unmapped read pairs were collected and used for *de novo* assembly. *De novo* assembly was performed using SPAdes v. 3.13.0 (42). Gene detection of the draft genome assemblies was performed using the DDBJ Fast Annotation and Submission Tool with default settings (43). The resulting assemblies were used for further comparative genome analysis.

### WTA extraction and chemical analysis.

Cell walls of *T. halophilus* were prepared as described previously (36) with some modifications. The cells were grown to the stationary phase in MRS-10 medium and harvested by centrifugation (8,000 × *g*, 5 min, 4°C). The cell pellets were washed with water, resuspended in 4% sodium dodecyl sulfate, boiled for 30 min, and incubated at room temperature overnight. Then, the suspension was boiled again for 10 min, and the pellets were collected by centrifugation, washed four times with water, and treated with DNase, RNase, and trypsin. After thorough washing with water, cell walls were pelleted by centrifugation and lyophilized. WTA was extracted by following the procedure of Bron et al. (44). Briefly, cell walls were treated with 10% TCA, and released WTA was precipitated by the addition of three volumes of ethanol for 24 h at 4°C. WTA was collected by centrifugation, washed with 70% ethanol twice, and lyophilized. To extract Rbo and Gro, 50 mg of WTA was hydrolyzed in 6 M HCl at 100°C for 3 h and neutralized with 4 M NaOH. As an internal standard, 20 μL of a 1,000-ppm solution of [1,1,2,3,3-D_5_]-glycerol (Cambridge Isotope Laboratories, Inc., Tewksbury, MA) was added to 200 μL of the neutralized hydrolysate. Samples were derivatized with benzoyl chloride according to the procedure of Bornø et al. (45). Derivatized Rbo and Gro in the hydrolysates were analyzed by LC/MS. LC was performed on an ACQUITY ultra-performance LC (UPLC) H-Class (Waters Corporation, Milford, MA). An ACQUITY UPLC ethylene bridged hybrid (BEH) C_18_ column (2.1 × 100 mm; Waters Corporation) was used at room temperature. Milli-Q water containing 10 mM ammonium formate (pH 3.5) was used as mobile phase A, and acetonitrile was used as mobile phase B. A gradient was used starting at 60% B and increasing to 90% within 7 min and reequilibrated at 60% B for 4.5 min followed by column washing at 90% B for 3 min. The flow rate was set at 0.3 mL/min. Three microliters of the sample was injected. MS analysis was performed on a Xevo G2-XS quadrupole-time-of-flight (Q-TOF) MS (Waters Corporation). Electrospray ionization was used at positive polarity at the following parameter settings: capillary voltage of 2.0 kV, sampling cone voltage of 40 V, source temperature of 120°C, and desolvation gas temperature of 450°C. The mass detection range was from *m*/*z* 150 to 1,000.

### Bacteriophage adsorption assay.

Phage adsorption by cell walls was measured according to the method of Baptista et al. (20). Cell walls were prepared as described above and resuspended at 10 μg/mL. Phages were mixed with cell walls and incubated at 30°C for 10 min. Control mixtures without cell walls were used to confirm the phage input in the experiment. To assess total adsorption, the mixture was centrifuged at 6,000 × *g* for 5 min at 4°C, and the supernatant was assayed for plaques using WJ7 as the indicator strain. Free phages (unbound phages) in the supernatants after centrifugation were measured. To assess irreversible adsorption, the mixture was diluted 100-fold in 10% NaCl, vortexed for 5 s, equilibrated for 5 min at room temperature, and centrifuged. Free phages (unbound and reversibly bound phages) in the supernatants were enumerated. For statistical analysis, the data were assessed by one-way analysis of variance (ANOVA) followed by a *post hoc* Tukey’s multiple-comparison test using R software (version 3.6.0; www.r-project.org). Statistical significance was considered at *P* < 0.05.

### Bioinformatics.

The DNA and amino acid sequences were analyzed using BLAST. The phylogenetic tree was constructed based on the alignment of the amino acid sequences from the major capsid protein of phiWJ7 and other *Rountreeviridae* phages. Their sequence data are available in the NCBI database. The amino acid sequences were aligned with Clustal X (version 2.1), and the neighbor-joining phylogenetic tree was drawn by NJplot (version 2.3) with bootstrap analysis (1,000 replicates).

### Data availability.

Illumina sequence reads of WJ7R1, WJ7R2, and phiWJ7 were deposited in the DDBJ Sequence Read Archive. The DRA accession numbers for WJ7R1, WJ7R2, and phiWJ7 are DRR311734, DRR311735, and DRR311536, respectively. Other sequence data were deposited in the DDBJ/EMBL/GenBank databases, with accession numbers LC638494 to LC638497 and LC640109 to LC640133.
