# Supplementary material for: Ribitol-Containing Wall Teichoic Acid of Tetragenococcus halophilus Is Targeted by Bacteriophage phiWJ7 as a Binding Receptor
Source: Microbiol Spectr. 2022 Mar 21;10(2):e00336-22. doi: 10.1128/spectrum.00336-22 (PMC9045211; doi:10.1128/spectrum.00336-22)
Supplement: SUPPLEMENTAL FILE 1 — Supplemental material. Download SPECTRUM00336-22_Supp_1_seq8.pdf, PDF file, 0.7 MB [file spectrum00336-22_supp_1_seq8.pdf]

Table S1. Putative ORFs in the phiWJ7 genome.

ORF	Coding region	length	Identity	Homology	
No.		(aa)	(%)	Probable function	Source
3	795-1319	174	44	ssDNA-binding protein	*Lactococcus* phage asccphi28
5	1873-3120	415	49	DNA packaging protein	*Staphylococcus* phage SLPW
6	3124-5427	767	45	DNA polymerase	*Staphylococcus* phage Andhra
9	5912-7075	387	70	phage capsid protein	*Syntrophomonadaceae* bacterium
10	7078-8091	337	47	upper collar protein	*Staphylococcus* phage Andhra
11	8051-8716	221	41	lower collar protein	*Staphylococcus* phage SA03-CTH2
12	8727-10649	640	34	putative phage structural protein	*Staphylococcus* phage PSa3
13	10665-11471	268	69	BppU family phage baseplate upper protein	*Tetragenococcus halophilus*
14	11471-11692	73	77	hypothetical protein	*Tetragenococcus halophilus*
15	11715-13463	582	46	phage tail protein	*Enterococcus faecalis*
16	13467-14978	503	42	peptidase	*Staphylococcus* phage Andhra
17	14991-15434	147	27	hypothetical protein	*Enterococcus gallinarum*

18	15424-15657	77	54	phage holin	unclassified *Enterococcus*
19	15654-16595	313	50	glycosyl hydrolase family 25	*Tetragenococcus halophilus*
20	16812-16630	60	83	hypothetical protein	*Tetragenococcus muriaticus*

Only the ORFs with significant homology are listed here.

Table S2. Relative efficiency of plating.

	Relative efficiency of plating		
Strains	phiWJ7	phiWJ7_2	phiWJ7_3
WJ7	1.0	1.0	1.0
WJ7R1	$<1.8\times10^{-9}$	1.1	1.6
WJ7R2	$<1.8\times10^{-9}$	0.9	1.6

Values are expressed as the mean (n=3).

Table S3. DNA sequences of the insertion sites and their flanking regions of IS*Teha3*, IS*Teha4* and IS*Teha5* on *tarIJL*.

IS transposed in each derivative	Flanking sequence at 5’ terminal	Insertion site (Duplicated target sequence)	Flanking sequence at 3’ terminal
IS*Teha3* in WJ7R1	GAATAATCAC	TAATCAA	TACATCAGTA
IS*Teha4* in WJ7R2	ATTTTATTTA	TTACTGA	TGTATTGATT
IS*Teha4* in WJ7R3	AATCAATACA	TCAGTAA	TAAATAAAAT
IS*Teha3* in WJ7R5	AACATCAAAA	TTACTAC	TCCTTATGAT
IS*Teha4* in WJ7R6	AGGGTATATT	TTATTTA	CTAAAACAGC
IS*Teha4* in WJ7R7	TATGTTTAAG	TAAATTGA	TATTAAACGA
IS*Teha4* in WJ7R8	GCTGTTTTAG	TAAATAA	AATATACCCT
IS*Teha4* in WJ7R9	GGTAAAATAA	TTACTTA	TTAAACCGCT
IS*Teha4* in WJ7R10	AGGGTATATT	TTATTTA	CTAAAACAGC
IS*Teha4* in WJ7R11	AATTTTTAGA	TTTAGAT	GGTAAACCGA
IS*Teha4* in WJ7R13	ATTTTATTTA	TTACTGA	TGTATTGATT
IS*Teha4* in WJ7R14	AATCAATACA	TCAGTAA	TAAATAAAAT

IS*Teha4* in WJ7R15	CGTAAAAATC	TCTAGAA	GTAATATAAT
IS*Teha4* in WJ7R16	ACACATAGAA	TCATAGAA	GAAATATCGG
IS*Teha4* in WJ7R17	AGCGGTTTAA	TAAGTAA	TTATTTTTAC
IS*Teha4* in WJ7R19	AGGGTATATT	TTATTTA	CTAAAACAGC
IS*Teha4* in WJ7R20	AACGGCTTAA	TTTATAA	TCCGTCTTAC
IS*Teha4* in WJ7R21	GCTGTTTTAG	TAAATAA	AATATACCCT
IS*Teha4* in WJ7R22	ATTTTATTTA	TTACTGA	TGTATTGATT
IS*Teha4* in WJ7R23	GAATTTTATA	TTCTTTA	TCTAAGCCTT
IS*Teha4* in WJ7R25	TTTTAAATAA	TTACAAAA	TTAATGATGA
IS*Teha4* in WJ7R26	ATTAATTTTG	TAATTAT	TTAAAATGTC
IS*Teha5* in WJ7R27	TAAAAACCTG	ATGAATTTT	ATATTCTTTA

Table S4. Amino acid sequence identity between the WTA synthesis genes in *S. aureus* COL and their homologs in *T. halophilus* NBRC 12172.

Gene	*S. aureus* COL		*T. halophilus* NBRC 12172		Identity (%)
tagO	SACOL0810	351 aa	TEH_07740	378 aa	41
mnaA	SACOL2103	375 aa	TEH_15300	370 aa	57
tagD	SACOL0698	132 aa	TEH_15990	131 aa	68
tagA	SACOL0693	254 aa	TEH_16230	244 aa	36
			TEH_16350	248 aa	36
tagB	SACOL0696	367 aa	TEH_16240	384 aa	25
			TEH_16360	384 aa	26
tarF	SACOL0239	389 aa	TEH_16170	1097 aa	37
			TEH_16220	395 aa	44
			TEH_16260	657 aa	47
			TEH_16310	1123 aa	31
			TEH_16340	393 aa	41
tarJ	SACOL0241	341 aa	-	-	-
tarI	SACOL0240	238 aa	-	-	-
tarL	SACOL0242	562 aa	-	-	-
tagG	SACOL0695	270 aa	TEH_15970	269 aa	34
tagH	SACOL0694	264 aa	TEH_15980	352 aa	48
lcpA	SACOL1398	327 aa	TEH_14040	380 aa	41
			TEH_15960	429 aa	29
			TEH_03890	310 aa	37

Identity (%) means amino acid similarity.

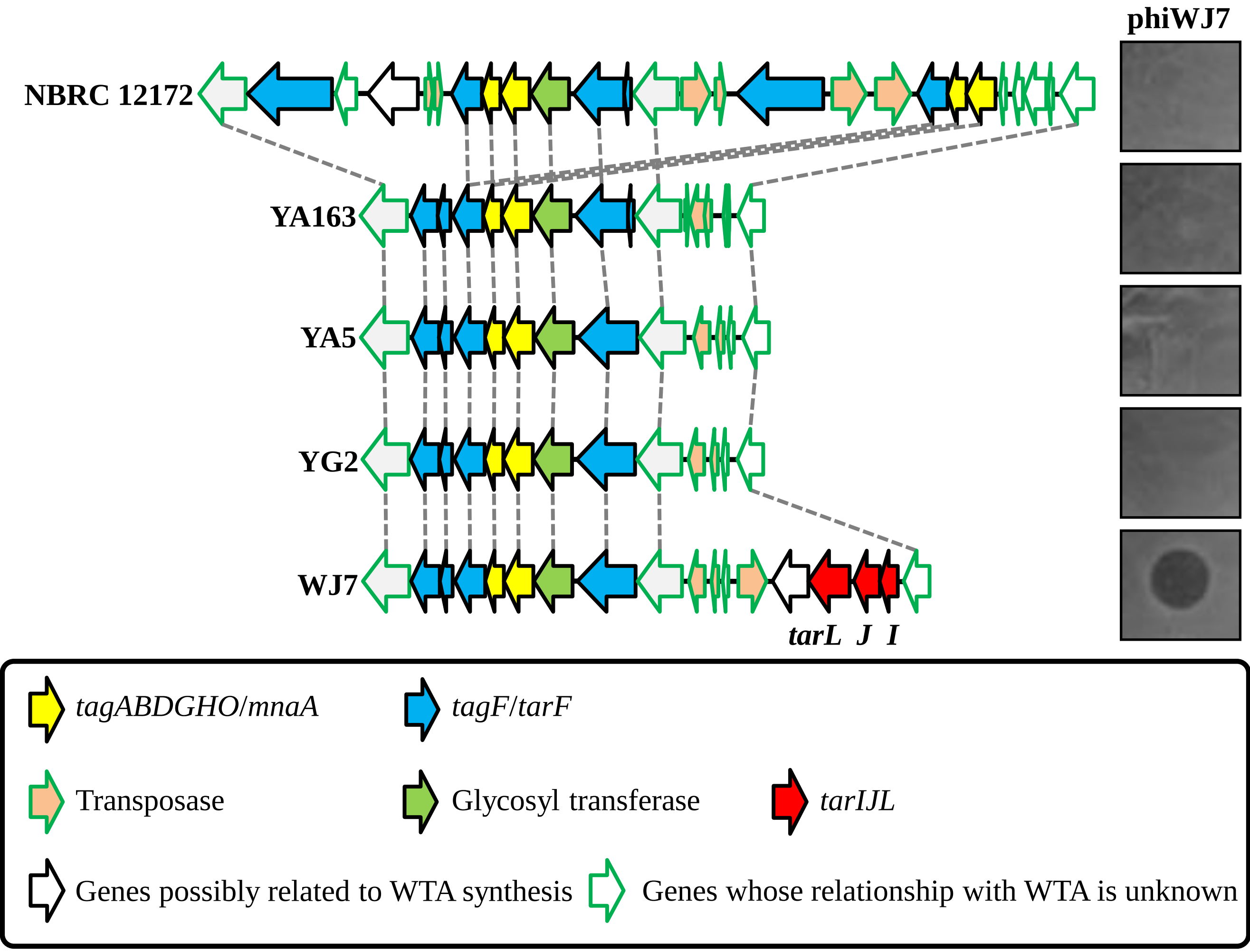

Figure S1: Comparison of *tagAB*-containing loci among five strains and their susceptibility to phiWJ7. Homologous ORFs with amino acid sequence identities above 90% are connected with gray dotted lines, except for transposases. phiWJ7 (4.3×10^7 PFU) was spotted on each host strain.

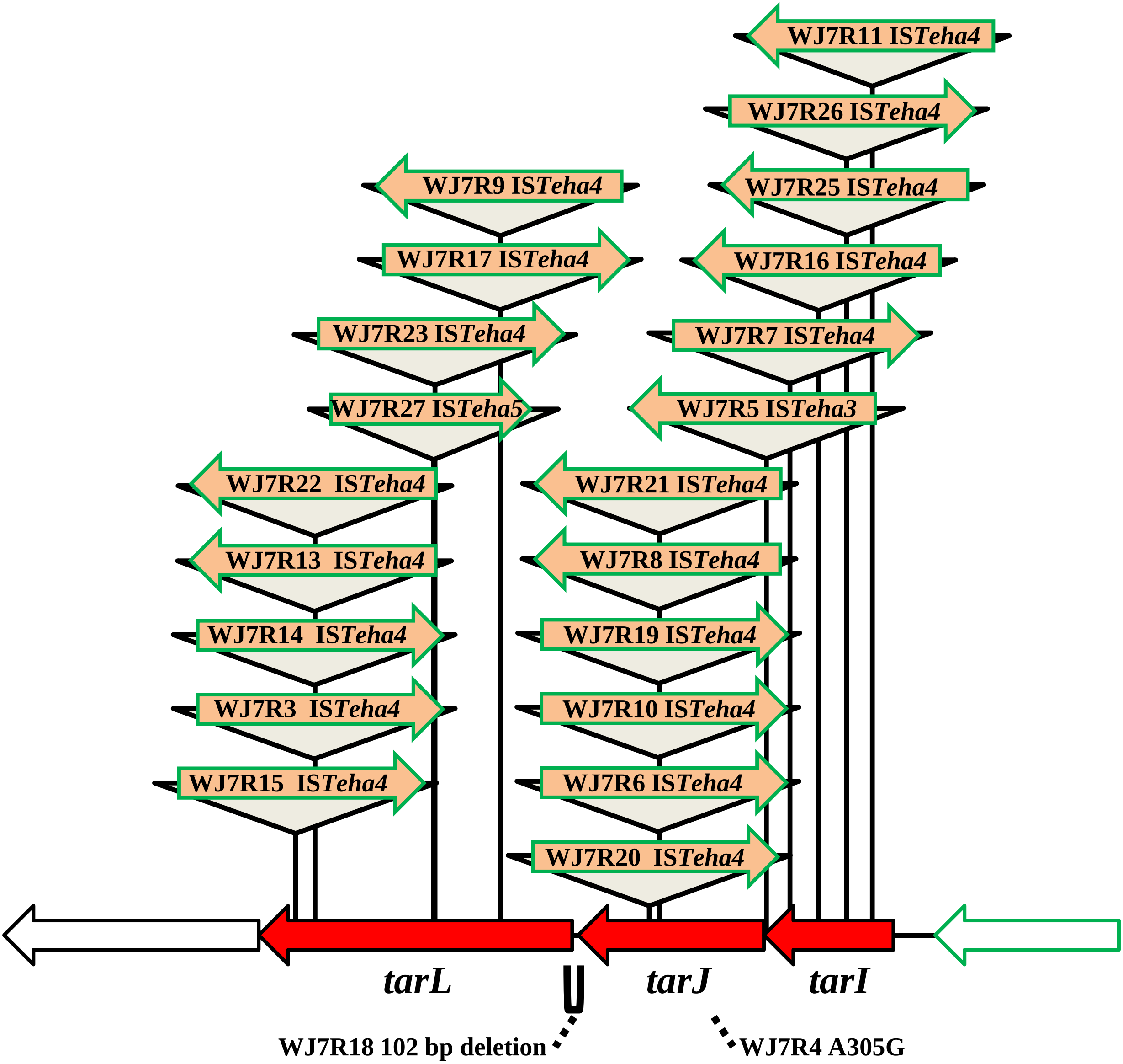

Figure S2: Mutations in the additionally acquired phiWJ7-insensitive derivatives on *tarIJL*.

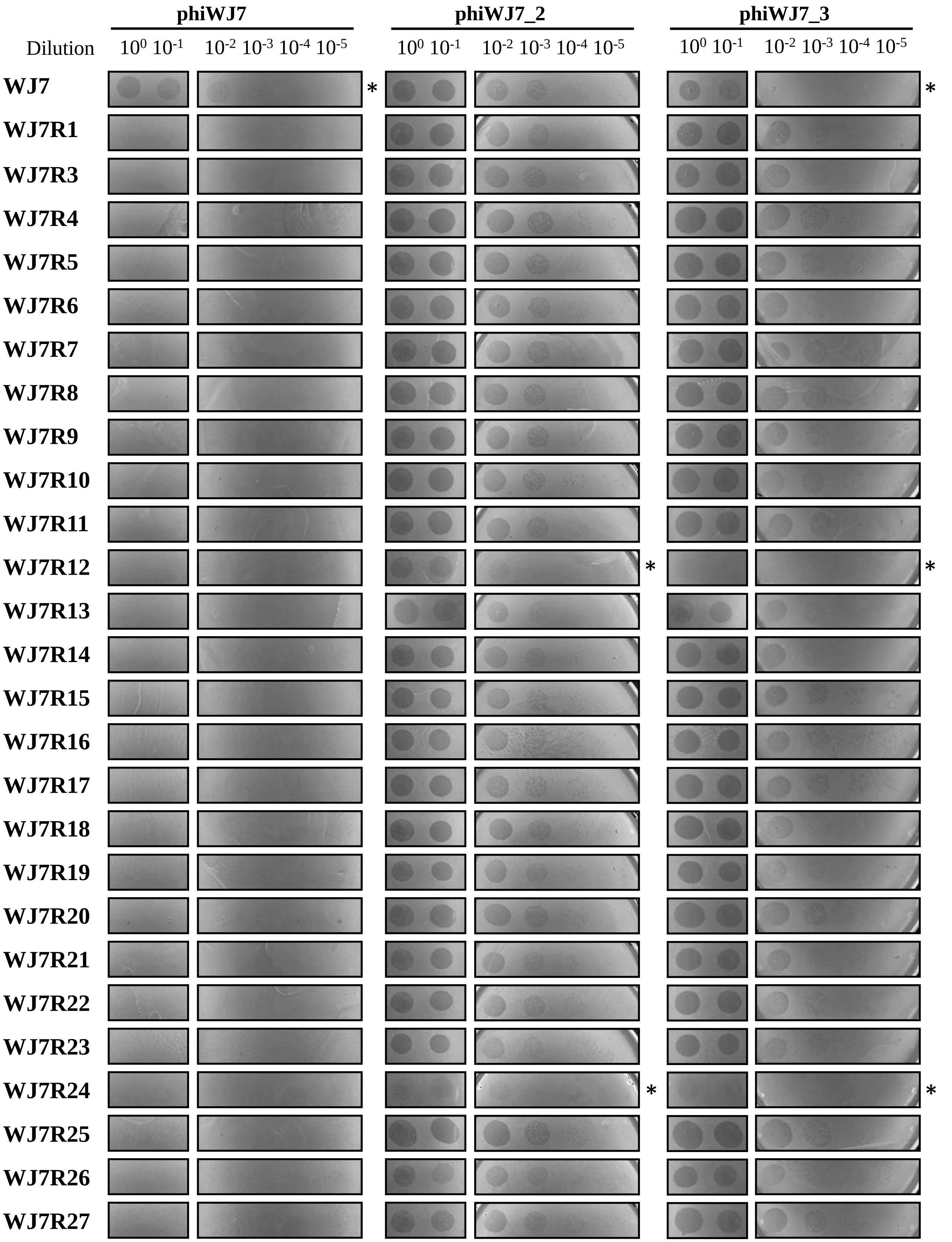
phiWJ7
phiWJ7_2
phiWJ7_3
Dilution
10^0 10^-1 10^-2 10^-3 10^-4 10^-5
WJ7
WJ7R1
WJ7R3
WJ7R4
WJ7R5
WJ7R6
WJ7R7
WJ7R8
WJ7R9
WJ7R10
WJ7R11
WJ7R12
WJ7R13
WJ7R14
WJ7R15
WJ7R16
WJ7R17
WJ7R18
WJ7R19
WJ7R20
WJ7R21
WJ7R22
WJ7R23
WJ7R24
WJ7R25
WJ7R26
WJ7R27
*

Figure S3: Phage susceptibility of the additionally acquired phiWJ7-insensitive derivatives. Asterisks on the right side of the panel indicate the apparent differences from WJ7R1. The various dilutions of a phage were spotted on the same plate of a strain, though it is divided.

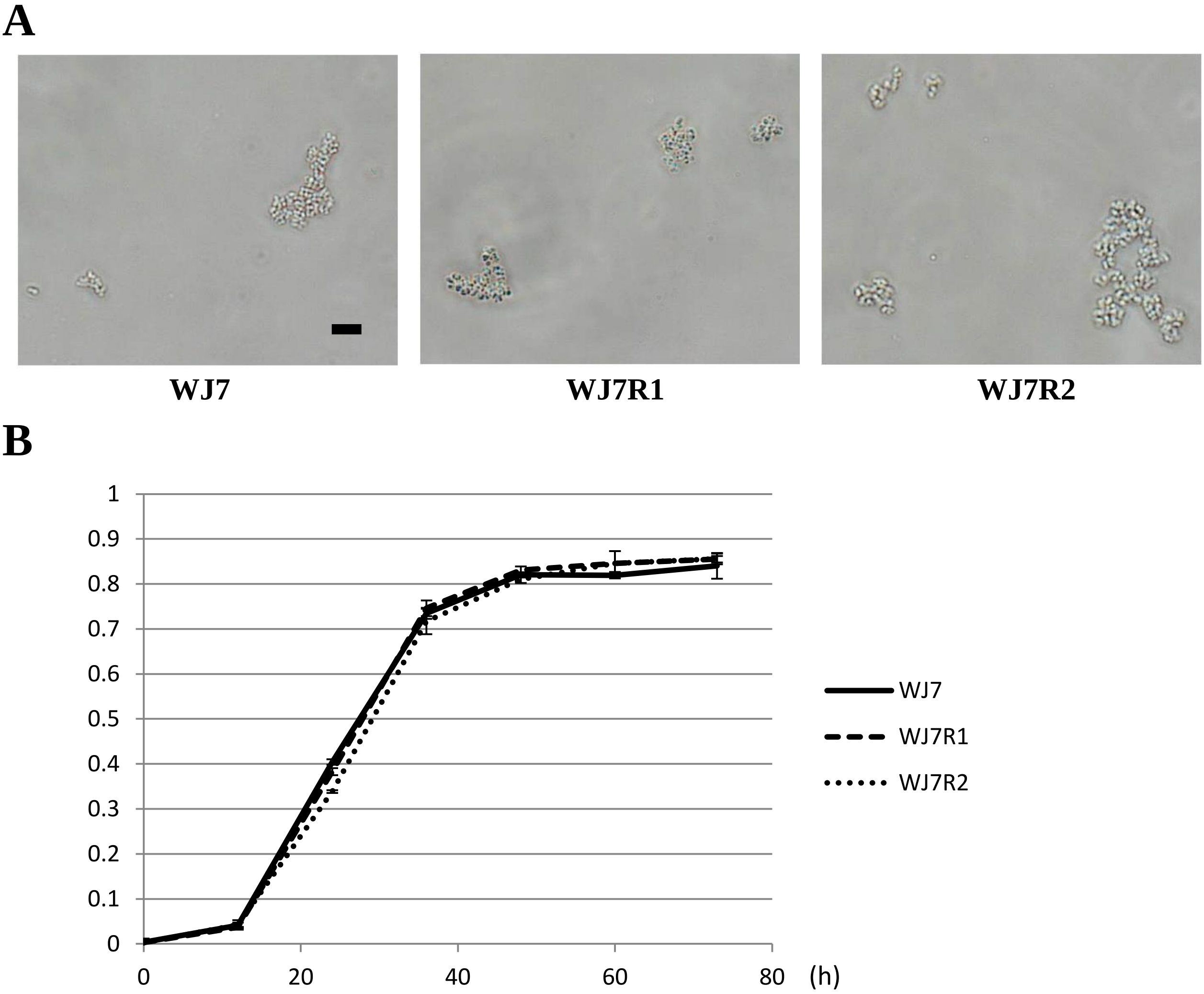

Figure S4: (A) Cell morphology of WJ7 and the derivatives. Black bar represents 10 μm. (B) Growth curve of WJ7 and the derivatives. Fully grown cultures of each strain were 100-fold diluted to fresh MRS-10 medium and OD_{660} of the cultures were measured periodically. Data are expressed as the mean with error bars representing ± SD (n=3).

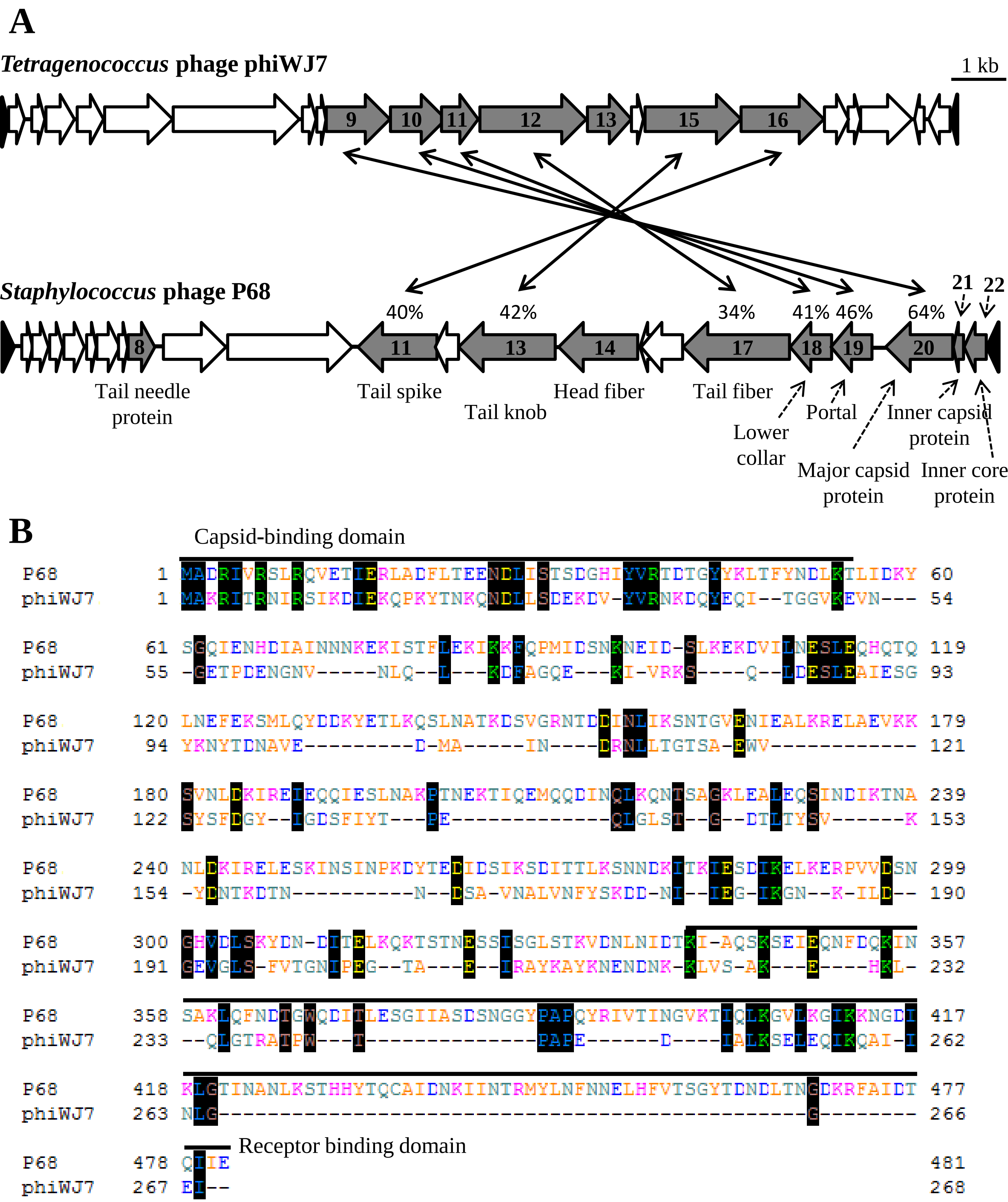
A
Tetragenococcus phage phiWJ7
1 kb
9
10
11
12
13
15
16
Staphylococcus phage P68
40%
42%
34%
41%
46%
64%
21
22
8
11
13
14
17
18
19
20
Tail needle protein
Tail spike
Tail knob
Head fiber
Tail fiber
Lower collar
Portal
Major capsid protein
Inner capsid protein
Inner core protein
B
Capsid-binding domain
P68 1 MADRIVRSLRQVETIERLADFLTEENDLISTSDGHIYVRTDTGYYKLTFYNDLKTLIDKY 60
phiWJ7 1 MAKRITRNIRSIKDIEKQPKYTNKQNDLLSDEKDV-YVRNKDQYEQI--TGGVKEVN--- 54
P68 61 SGQIENHDIAINNNKEKISTFLEKIKKFQPMIDSNKNEID-SLKEKDVILNESLEQHQTQ 119
phiWJ7 55 -GETPDENGNV-----NLQ--L---KDFAGQE---KI-VRKS----Q--LDESLEAIESG 93
P68 120 LNEFEKSMLQYDDKYETLKQSLNATKDSVGRNTDDINLIKSNTGVENIEALKRELAEVKK 179
phiWJ7 94 YKNYTDNAVE---------D-MA-----IN----DRNLLTGTSA-EWV------------ 121
P68 180 SVNLDKIREIEQQIESLNAKPTNEKTIQEMQQDINQLKQNTSAGKLEALEQSINDIKTNA 239
phiWJ7 122 SYSFDGY--IGDSFIYT---PE-------------QLGLST--G--DTLTYSV------K 153
P68 240 NLDKIRELESKINSINPKDYTEDIDSIKSDITTLKSNNDKITKIESDIKELKERPVVDSN 299
phiWJ7 154 -YDNTKDTN----------N--DSA-VNALVNFYSKDD-NI--IEG-IKGN--K-ILD-- 190
P68 300 GHVDLSKYDN-DITELKQKTSTNESSISGLSTKVDNLNIDTKI-AQSKSEIEQNFDQKIN 357
phiWJ7 191 GEVGLS-FVTGNIPEG--TA---E--IRAYKAYKNENDNK-KLVS-AK---E----HKL- 232
P68 358 SAKLQFNDTGWQDITLESGIIASDSNGGYPAPQYRIVTINGVKTIQLKGVLKGIKKNGDI 417
phiWJ7 233 --QLGTRATPW---T--------------PAPE------D----IALKSELEQIKQAI-I 262
P68 418 KLGTINANLKSTHHYTQCAIDNKIINTRMYLNFNNELHFVTSGYTDNDLTNGDKRFAIDT 477
phiWJ7 263 NLG--G-------- 266
Receptor binding domain
P68 478 QIIE 481
phiWJ7 267 EI-- 268

Figure S5: (A) Schematic representation of the phiWJ7 genome and the P68 genome. Phage structural proteins are gray-shaded. Percentage shows the amino acid identities between phiWJ7 and P68. The numbers on the arrows indicate the ORF number. (B) Alignment of the head fiber protein (ORF14) of P68 and ORF13 of phiWJ7. The capsid binding domain and the receptor binding domain of P68 head fiver are indicated by overlines.
